# Correlation of Sarcopenia with Coronary Artery Disease Severity and Pericoronary Adipose Tissue Attenuation: A Coronary CT Study

**DOI:** 10.3390/tomography10110128

**Published:** 2024-10-30

**Authors:** Domenico Albano, Caterina Beatrice Monti, Giovanni Antonio Risoleo, Giacomo Vignati, Silvia Rossi, Edoardo Conte, Daniele Andreini, Francesco Secchi, Stefano Fusco, Massimo Galia, Paolo Vitali, Salvatore Gitto, Carmelo Messina, Luca Maria Sconfienza

**Affiliations:** 1 IRCCS Istituto Ortopedico Galeazzi, 20161 Milan, Italyio@lucasconfienza.it (L.M.S.); 2 Dipartimento di Scienze Biomediche, Chirurgiche ed Odontoiatriche, Università Degli Studi di Milano, 20122 Milan, Italy; 3 Postgraduate School of Diagnostic and Interventional Radiology, Università Degli Studi di Milano, 20122 Milan, Italysilvia.rossi1@unimi.it (S.R.); 4 Radiology Department, Fatebenefratelli Hospital, ASST Fatebenefratelli Sacco, Piazza Principessa Clotilde 3, 20121 Milan, Italy; 5 Division of University Cardiology and Cardiac Imaging, IRCCS Ospedale Galeazzi-Sant’Ambrogio, 20157 Milan, Italy; edoardo.conte@grupposandonato.it (E.C.);; 6 Department of Biomedical and Clinical Sciences, University of Milan, 20157 Milan, Italy; 7 Unit of Cardiovascular Imaging, IRCCS MultiMedica, Sesto San Giovanni, 20099 Milan, Italy; 8 Dipartimento di Scienze Biomediche per la Salute, Università Degli Studi di Milano, 20122 Milan, Italy; 9 Section of Radiology, Department of Biomedicine, Neuroscience and Advanced Diagnostics (BiND), University Hospital “Paolo Giaccone”, Via del Vespro 129, 90127 Palermo, Italy; 10 U.O.C. Radiodiagnostica, ASST Centro Specialistico Ortopedico Traumatologico Gaetano Pini-CTO, 20122 Milan, Italy

**Keywords:** sarcopenia, pericoronary adipose tissue, coronary artery disease, CAD-RADS, coronary computed tomography angiography

## Abstract

Objective: To investigate the association between sarcopenia, as appraised with CT-derived muscle metrics, and cardiovascular status, as assessed via coronary CT angiography (CCTA) using the Coronary Artery Disease-Reporting and Data System (CAD-RADS) and with pericoronary adipose tissue (pCAT) metrics. Methods: A retrospective observational study conducted on patients who underwent CCTA. The cross-sectional area (CSA) and attenuation values of the paravertebral muscles at the T8 level and the pectoralis major muscles at the T6 level were measured. The patient height was employed for the normalization of the skeletal muscle CSA. The pCAT attenuation around the coronary arteries was assessed, and the CAD severity was graded using the CAD-RADS reporting system. Regression analyses were performed to assess the impact of demographics, clinical factors, and CT variables on the CAD-RADS and pCAT. Results: A total of 220 patients were included (132 males, median age 65 years). Regression analyses showed the associations of CAD with age and sex (*p* < 0.001). Familiarity with CAD was related to the left anterior descending artery pCAT (*p* = 0.002) and circumflex artery pCAT (*p* = 0.018), whereas age was related to the left anterior descending artery pCAT (*p* = 0.032). Weak positive correlations were found between the lower muscle density and lower pCAT attenuation (ρ = 0.144–0.240, *p* < 0.039). Conclusions: This study demonstrated weak associations between the sarcopenia indicators and the cardiovascular risk, as assessed by the CAD severity and pCAT inflammation. However, these correlations were not strong predictors of CAD severity, as age and traditional cardiovascular risk factors overshadowed the impact of sarcopenia in the cardiovascular risk assessment.

## 1. Introduction

Sarcopenia has been defined as a condition characterized by progressive and generalized loss of muscle mass and function [1]. The prevalence of this syndrome has been on the rise worldwide due to an ever-aging population [2]. Recently, sarcopenia has become a hot topic, with a considerable amount of the literature revealing its clinical impact on several conditions. As a matter of fact, sarcopenia is acknowledged as an unfavorable predictor in cancers [3,4], trauma [5,6], major surgery [7,8], and even infectious diseases like COVID-2019 [9]. Assessing sarcopenia entails a thorough examination, relying on clinical assessments (e.g., gait speed test), questionnaires like the SARC-F, bioelectrical impedance analysis, and possible use of imaging examinations such as dual-energy X-ray absorptiometry (DXA), computed tomography (CT), magnetic resonance, and ultrasound [10,11,12]. Whole-body DXA is regarded as the reference standard imaging technique for the accurate identification of sarcopenic patients. It is routinely used in clinical practice, with well-established cut-off values [13]. CT has been widely used as an opportunistic tool for retrospective and prospective evaluation of sarcopenia in patients undergoing imaging examinations for various reasons [9,12]. Indeed, data obtained from muscle segmentation in a single cross-sectional image have been deemed highly precise in estimating body composition and the overall distribution of skeletal muscle [14,15]. This has made CT an optimal tool for research studies on sarcopenia.

The pathophysiology of sarcopenia encompasses several factors, such as the decline in muscle mass associated with aging, changes in vitamin and protein consumption, and reduced levels of physical activity but also a pro-inflammatory status [16]. The latter is also involved in the pathophysiology of coronary artery disease (CAD) as a major driving force in the initiation of coronary plaques, their unstable progression, and eventual disruption, in conjunction with other widely recognized risk elements for CAD, like hypertension, diabetes, smoking, hypercholesterolemia, and familiarity [17,18,19]. CAD is increasingly often assessed noninvasively through coronary CT angiography (CCTA) due to its impressive spatial and temporal resolution. Recently, the updated version of the CAD-RADS reporting system has been published to provide a standardized classification of CAD on CCTA, evaluating the coronary stenosis, plaque burden and modifiers such as patients having undergone prior procedures, along with evidence-based recommendations for patient management [20]. Moreover, novel applications such as photon-counting CT could potentially unlock new potential for CCTA, adding to the list of biomarkers that can be derived from such techniques [21].

As abovementioned, inflammation plays a crucial role in the initiation and advancement of CAD. Recently, epicardial adipose tissue, especially pericoronary adipose tissue (pCAT), has been discovered to be a noninvasive biomarker of vascular inflammation that can be evaluated on CT scans [22,23]. This is due to the potential influence of cytokines released from inflamed vessels on the composition of pCAT [24]. Increased pCAT attenuation values, indicating inflammation, have been linked to increased morbidity and mortality [25].

Thus, CCTA could be regarded as an optimal imaging examination to assess patients’ muscle status by measuring the cross-sectional area and attenuation values of the paravertebral (PV) and pectoralis major (PM), altogether appraising the CAD-RADS score and pCAT characteristics. Indeed, to date, while there is increased evidence of the role of pCAT as a biomarker of inflammatory changes, especially in CAD patients, the potential link between sarcopenia and CAD has received limited investigation. Prior research has yielded encouraging findings, suggesting that sarcopenia might serve as an indicator of subclinical atherosclerosis and a strong predictor of significant adverse cardiovascular events. Previous studies have linked sarcopenia to cardiovascular disease, finding promise in such a regard [26,27]. Nevertheless, conflicting outcomes have arisen regarding its connection with the CAD burden and complexity [28,29,30,31,32,33]. Therefore, the aim of this study was to evaluate the association of CAD-RADS and the pCAT metrics assessed on CCTA with CT-derived muscle metrics.

## 2. Materials and Methods

### 2.1. Study Design

This study is a retrospective observational investigation conducted at a single center. Approval from the Institutional Review Board was obtained (RETRORAD protocol, Ospedale San Raffaele Ethical Committee), and all the patients had provided written informed consent for the potential use of the results of their examination for scientific purposes. After matching the imaging and clinical data, our database was anonymized to remove any connections between the data and patients’ identity according to the General Data Protection Regulation.

We included all the consecutive patients who were underwent CCTA at the Radiology Department of IRCCS Ospedale Galeazzi-Sant’Ambrogio to be subjected to CCTA between January and April 2023, regardless of clinical indication. We excluded all those patients where the segmentation of the PV or MG skeletal muscle was not feasible (e.g., motion artifacts, spine implants, prosthesis, muscles not completely included in CCTA scans) and those with CT images of poor quality due to artifacts that hindered the proper assessment of CAD (blurring, stairstep or banding). Additionally, we excluded patients with incomplete clinical data, or those with chronic neuromuscular disorders that impact muscular condition over time (e.g., Duchenne dystrophy), or complex congenital cardiovascular diseases, which could hinder the CAD evaluation. Clinical and demographic data were extracted from the CCTA reports, as, in accordance with the usual procedures of our department, height, weight, comorbidities, and risk factors for CAD are consistently reported in such documents.

### 2.2. CCTA Protocol

All the CCTA scans were performed using a single-source (16 cm-detector) CT scanner (Revolution CT 512, GE Medical, Milwaukee, MI, USA) with ECG-gated acquisitions, using the automatic tube voltage and current selection systems, with a reference tube voltage of 100 kVp and reference tube current-time product of 370 mAs/rotation. All the CCTA scans were synchronized with the first pass of the contrast agent using the visual bolus-tracking method. Iodinated contrast medium (Iomeron 400 mg/mL, Bracco Imaging Italia, Milan, Italy) was administered using a biphasic injection scheme: 50 mL contrast agent, followed by 30 mL of saline solution. In all the patients, premedication with a metoprolol 5 mg iv bolus up to 20 mg for heart rate control and sublingual nitrates were administered prior to the CCTA acquisition, except when contraindicated (e.g., patients with glaucoma, asthma, allergic to specific drugs). Multiphase images were reconstructed in standard fashion at every 10% of the R-R interval (0–90%), at a slice thickness of 0.625 mm, with an increment of 0.5 mm, using a smooth kernel and an iterative reconstruction algorithm.

### 2.3. CCTA Image Evaluation

**Figure 1:**
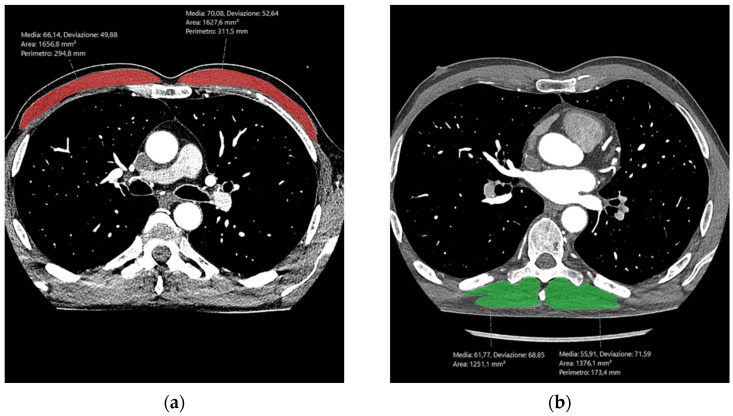
Example of the segmentation of the pectoralis major muscles at the T6 level (red areas, (**a**)) and paravertebral skeletal muscle at the T8 level (green areas, (**b**)). For each muscle, the mean attenuation in Hounsfield units and the cross-sectional area (CSA) were measured.

Segmentation of the skeletal muscle was performed by one radiologist with 10 years of experience in musculoskeletal imaging using the picture archiving and communications system viewer tools at our institution [34,35]. The measurement of the skeletal muscle area was conducted at the T8 pedicle level, noting the attenuation in Hounsfield units and the cross-sectional area (CSA) of the paravertebral skeletal muscles on both sides of the spine, including the erector spinae muscle, longissimus thoracis muscle, spinalis thoracis muscle, and iliocostalis lumborum muscle. Such a level was chosen due to its widespread representation throughout the study sample, along with the fact that measurements at T6–T8 have been validated to assess sarcopenia [36]. Also, the attenuation values and CSA of the right and left PM muscles were measured on a single axial CCTA slice of CCTA at the T6 level (Figure 1). Then, the patient height was employed for the normalization of the skeletal muscle CSA (nCSA) and to derive the PV and PM indexes, dividing the CSA by the patient height^2^.

Regarding the evaluation of CAD, one radiologist with 5 years of experience in cardiovascular imaging graded the CCTA scans using the CAD-RADS reporting system, a grading scale based on the coronary stenosis severity and plaque burden, as follows: 0, no evidence of stenosis; 1, minimal stenosis (1–24%); 2, mild stenosis (25–49%); 3, moderate stenosis (50–69%); 4, severe stenosis, divided in two groups: 4A (70–99%) and 4B-Left main > 50% or 3-vessel obstructive disease; 5, 100% total occlusion [37]. The CAD-RADS categories can be complemented by modifiers to indicate the presence of stents (Ss), grafts (Gs), and high-risk plaque (HRP).

**Figure 2:**
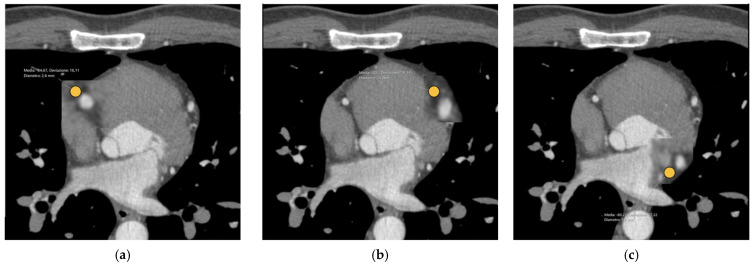
Example of the pericoronary adipose tissue segmentation on the post-contrast CCTA images. The regions of interest (ROIs—orange circles) are placed in the pCAT surrounding the right coronary artery (**a**), the left anterior descending artery (**b**), and circumflex artery (**c**).

The two raters also measured the pCAT attenuation values on the post-contrast CCTA image, placing regions of interest (ROIs) in the pCAT surrounding the left anterior descending artery (LAD), circumflex artery (CX), and right coronary artery (RCA), as reported in Figure 2.

### 2.4. Statistical Analysis

Statistical analyses were performed using Python v. 3.7, and *p*-values ≤ 0.05 were considered to indicate statistical significance [34]. Categorical variables were reported as numbers and percentages, while continuous variables were reported as means and standard deviations (SDs) or as medians and interquartile ranges (IQRs) according to their distribution, as assessed by visual analyses paired with the Shapiro–Wilk test. Regression analyses, either linear or logistic with regards to the endpoint variable, were conducted to assess the impact of demographical, clinical and CT variables on the CAD-RADS and pCAT. Correlations between the muscle CT metrics and CAD-RADS or pCAT were appraised with Pearson’s r or Spearman’s ρ, according to the data distributions. Considering that for each of the four endpoint variables, namely CAD-RADS and pCAT on the three coronary arteries, three correlations were computed, with the CSA, nCSA and density, respectively, the *p*-value threshold for statistical significance was divided by 3 as per the Bonferroni correction, leading to *p* ≤ 0.017.

## 3. Results

**Figure 3:**
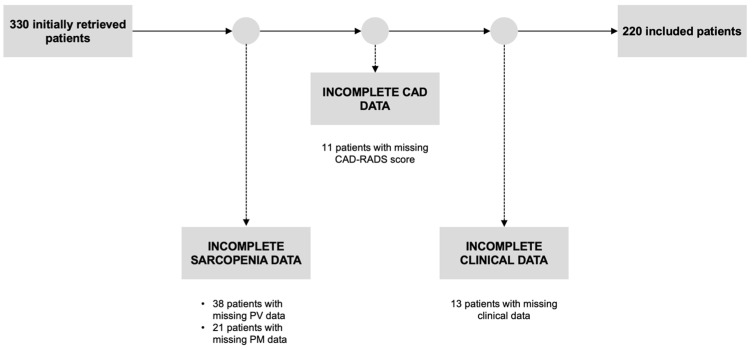
Flowchart depicting the patient selection process. PV: paravertebral muscle; PM: pectoralis major muscle; CAD: coronary artery disease; CAD-RADS: CAD Reporting and Data System.

Starting from 303 patients initially retrieved, who had undergone CCTA at our institution between January and April 2023, 38 were excluded due to missing PV data, 21 due to missing PM data, 11 were excluded as the CAD-RADS score was not computable due to CCTA artifacts, and 13 presented with incomplete clinical data. Eventually, this led to 220 included patients, 132 (60%) of whom were males, with a median age of 65 years (IQR 58–72 years). A flowchart depicting the patient selection process is shown in Figure 3.

Overall, the median PV density was 38 HU (IQR 26–48 HU), the median PV CSA was 18.19 cm^2^ (IQR 13.87–22.77 cm^2^), and the median PV nCSA was 21.49 cm (16.34–26.84 cm). The patients’ median CAD-RADS score was 2 (1–3), whereas the median pCAT density was −100 HU (IQR −109–−88 HU) around the LAD, −99 HU (IQR −113–−83 HU) around the CX, and −101 HU (IQR −112–−89 HU) around the RCA. Age and sex yielded a significant impact on the CAD-RADS score (*p* < 0.001). Concerning pCAT, age (*p* = 0.032) had a significant impact on the LAD pCAT, whereas familiarity with CAD had a significant impact on the LAD pCAT (*p* = 0.002) and CX pCAT (*p* = 0.018). Demographic data, along with the results from the regression analyses, are shown in Table 1.

Concerning the bivariate correlations, the PV density (ρ = 0.235 *p* < 0.001), PM density (ρ = 0.220 *p* = 0.001), PM CSA (ρ = 0.240 *p* < 0.001), and PM nCSA (ρ = 0.229 *p* < 0.001) displayed weak, albeit significant, positive correlations with the LAD pCAT. Moreover, the PV CSA and PM CSA displayed weak, borderline significant, positive correlations with the LAD pCAT (ρ = 0.150 *p* = 0.026 and ρ = 0.140 *p* = 0.039, respectively), and CX pCAT (ρ = 0.147 *p* = 0.029 and ρ = 0.144 *p* = 0.032, respectively). All the correlations between the CT muscle metrics and the cardiovascular biomarkers are displayed in Table 2.

## 4. Discussion

Sarcopenia is a serious condition, which is related to increased morbidity and mortality, not just in the elderly but also among those with cardiovascular disease [35], a population where the CCTA numbers are on the rise due to its potential in improving patient outcomes [36].

The results from our study indicate the presence of a very weak positive correlation between the PV density and the LAD pCAT (ρ = 0.235 *p* < 0.001), implying that a lower muscle density, indicating sarcopenia, is related to lower adipose tissue attenuation. As the ROIs were not centered on the vessels, but rather in the further pCAT, this could be explained by the fact that lower pCAT attenuation away from the coronary artery may indicate the presence of pericoronary inflammation, which leads to the presence of a steeper pCAT attenuation gradient [24,37]. Similarly, the very weak, albeit significant, positive correlations between the PM muscle metrics, namely the density, CSA and nCSA, and the LAD pCAT (ρ = 0.220 *p* = 0.001, ρ = 0.240 *p* < 0.001, and ρ = 0.229 *p* < 0.001, respectively) indicate that decreased PM muscle density and area, again related to sarcopenia, are, too, linked to lower adipose tissue attenuation. The same may be true for the borderline correlations, which still indicate the same trend for muscle density and pCAT. Overall, the lower attenuation of the adipose tissue further away from the coronary arteries may also be related to an overall whitening of the epicardial adipose tissue, which can be seen in patients with a heightened cardiovascular risk, who are at higher odds of developing CAD or other conditions such as atrial fibrillation [38]. Indeed, in physiologic conditions, epicardial adipose tissue is a beige type of adipose tissue, which can either display thermogenic proprieties, as brown adipose tissue does, or reduce its metabolic activity, reverting to white adipose tissue, which is linked to obesity, metabolic syndrome and aging [39,40,41].

The fact that the CT muscle metrics are not independent predictors of the CAD-RADS scores or pCAT values may be explained by the fact that sarcopenia, related to a decrease in muscle mass and density, is strongly associated with age, sex and cardiovascular risk factors, which are in turn stronger predictors of cardiovascular disease [42]. Indeed, age and familiarity with CAD are among the strongest independent predictors of cardiovascular disease, with their impact most likely overshadowing that of sarcopenia in forecasting CAD [43].

Previous works on chest CT have shown correlations between the CT-derived muscle metrics and outcomes related to cardiovascular disease. For instance, a work by Hathaway et al. [44] observed a link between the CT-derived PM muscle metrics obtained through artificial intelligence methods and the incidence of heart failure in a subclinical cardiovascular disease population. Another study by Teigen et al. [45] showed a correlation between the preoperative PM metrics and mortality after the implantation of left ventricular assist devices. Such findings may be explained by the fact that sarcopenia is indeed related to worse patient outcomes also related to cardiovascular disease [46]. However, as the presence of CAD indicated by a higher CAD-RADS score or variations in pCAT is not directly related to longer-term patient outcomes, correlations between the muscle metrics and the biomarkers of cardiovascular disease could not be observed in our study population. Most likely, while sarcopenia is associated with patient outcomes, both short- and long-term, its transverse correlations with individual cardiovascular risk factors are less prominent, even though both entities may be predictive of long-term events [47].

While the results from our work may suggest that the sarcopenia metrics are not significant lone predictors of the cardiovascular risk, with particular reference to CAD, the fact that such information is easily available as opportunistic biomarkers in patients undergoing CCTA may still prove useful to a certain extent [48]. Indeed, obtaining the PV or PM metrics requires only a small amount of time for muscle segmentation, which can also be performed with the aid of artificial intelligence applications, and may provide information that could be beneficial, especially if clinical data were missing, as identifying sarcopenia would still relate to the overall morbidity and mortality [49]. Moreover, despite the correlations being weak, sarcopenia is still related to the cardiovascular metrics, especially the pCAT density, to a certain extent, possibly providing some insight concerning patient inflammation and metabolic status. Hence, assessing sarcopenia, even on CCTA scans, may prove useful from a clinical standpoint, even though no strong relationship may exist with the cardiovascular risk alone.

Our study presents some limitations. First and foremost, it is a retrospective analysis of a small population, which may also suffer from some sort of selection bias due to the single-institution nature of the work. Hence, further studies with larger, more widely varied samples are warranted to better determine the role of skeletal muscle evaluation from CCTA. Moreover, the patients included in our study were relatively young and were referred to a secondary cardiological center; hence, they may present lower degrees of sarcopenia and cardiovascular risk factors (most of all, smoking) compared to the general population. This might have led to the lack of significant correlations between the CT muscle metrics and the cardiovascular risk biomarkers. Last, we did not assess the role of the assessment of sarcopenia from CCTA specifically in patients referred for procedures such as transcatheter aortic valve replacement or left atrial appendage closure, especially in the preoperative settings. Future studies could be aimed at performing such evaluations to better define the role of such analyses in the preprocedural setting.

## 5. Conclusions

In conclusion, the results from our study show that the muscle metrics derived from CCTA are related to the cardiovascular risk, albeit weakly, linking the cardiovascular risk and sarcopenia. Still, traditional risk factors have a higher impact on the overall cardiovascular risk evaluation. As such, these metrics might be considered when other, more robust clinical/imaging parameters are not available. Further studies among larger populations are warranted to review the role of skeletal muscle evaluation from CCTA.

## Figures and Tables

**Table 1:** Results from the regression analyses in patients with measurements for paravertebral muscle. CSA: cross-sectional area; nCSA: normalized cross-sectional area; HU: Hounsfield Units. * denotes statistical significance; pCAD-RADS: *p*-value for CAD-RADS score; pROI LAD: *p*-value for the region of interest on the left anterior descending artery; pROI CX: *p*-value for the circumflex artery; pROI CDX: *p*-value for the right coronary artery.

	n = 220	pCAD-RADS	pROI LAD	pROI CX	pROI CDX
Age	65 (58–72)	<0.001 *	0.032 *	0.880	0.312
Males (n, %)	132 (60)	<0.001 *	0.499	0.482	0.704
Height (cm)	170 (163–174)	0.866	0.764	0.250	0.743
Weight (kg)	75 (68–84)	0.073	0.081	0.138	0.476
Systolic pressure (mmHg)	130 (120–140)	0.737	0.362	0.171	0.916
Diastolic pressure (mmHg)	80 (75–80)	0.970	0.649	0.402	0.689
Dyslipidemia (n, %)	48 (22)	0.613	0.595	0.146	0.494
Hypertension (n, %)	42 (19)	0.118	0.162	0.105	0.991
Familiarity (n, %)	22 (10)	0.114	0.002 *	0.018 *	0.099
Prior AMI (n, %)	39 (18)	0.183	0.882	0.741	0.252
Smoking (n, %)	16 (7)	0.726	0.125	0.240	0.894
Diabetes (n, %)	9 (5)	0.077	0.915	0.530	0.710
PV density (HU)	38 (26–48)	0.954	0.900	0.337	0.768
PV CSA (cm^2^)	18.19 (13.87–22.77)	0.976	0.884	0.534	0.894
PV nCSA (cm)	21.49 (16.34–26.84)	0.842	0.632	0.335	0.974
PM density (HU)	46 (36–55)	0.213	0.463	0.133	0.833
PM CSA (cm^2^)	13.07 (9.51–17.57)	0.298	0.385	0.319	0.743
PM nCSA (cm)	4.62 (3.37–6.07)	0.442	0.570	0.349	0.881

**Table 2:** Correlations between the CT-derived muscle metrics for the paravertebral (PV) and pectoralis major (PM) muscles and the cardiovascular biomarkers, namely the CAD-RADS score and pericoronary adipose tissue (pCAT) surrounding the left anterior descending (LAD), circumflex (CX) and right (RCA) coronary arteries. CSA: cross-sectional area; nCSA: normalized cross-sectional area; HU: Hounsfield units. * denotes statistical significance; ** denotes borderline statistical significance.

		pCAT LAD (HU)	pCAT CX (HU)	pCAT RCA (HU)
PV	Density (HU)	ρ = 0.235 *p* < 0.001 *	ρ = 0.015 *p* = 0.822	ρ = −0.109 *p* = 0.871
	CSA (cm^2^)	ρ = 0.150 *p* = 0.026 **	ρ = 0.147 *p* = 0.029 **	ρ = 0.077 *p* = 0.257
	nCSA (cm)	ρ = 0.140 *p* = 0.039 **	ρ = 0.144 *p* = 0.032 **	ρ = 0.070 *p* = 0.301
PM	Density (HU)	ρ = 0.220 *p* = 0.001 *	ρ = 0.095 *p* = 0.160	ρ = 0.090 *p* = 0.186
	CSA (cm^2^)	ρ = 0.240 *p* < 0.001 *	ρ = 0.073 *p* = 0.280	ρ = 0.122 *p* = 0.071
	nCSA (cm)	ρ = 0.229 *p* < 0.001 *	ρ = 0.077 *p* = 0.258	ρ = 0.126 *p* = 0.062

## Data Availability

Data are available from the corresponding author upon request.

## References

[1] Cruz-Jentoft A.J., Landi F., Schneider S.M., Zúñiga C., Arai H., Boirie Y., Chen L.-K., Fielding R.A., Martin F.C., Michel J.-P. (2014). Prevalence of and interventions for sarcopenia in ageing adults: A systematic review. Report of the International Sarcopenia Initiative (EWGSOP and IWGS). Age Ageing.

[2] Cruz-Jentoft A.J., Baeyens J.P., Bauer J.M., Boirie Y., Cederholm T., Landi F., Martin F.C., Michel J.-P., Rolland Y., Schneider S.M. (2010). Sarcopenia: European consensus on definition and diagnosis. Age Ageing.

[3] Shachar S.S., Williams G.R., Muss H.B., Nishijima T.F. (2016). Prognostic value of sarcopenia in adults with solid tumours: A meta-analysis and systematic review. Eur. J. Cancer.

[4] Chang K.-V., Chen J.-D., Wu W.-T., Huang K.-C., Hsu C.-T., Han D.-S. (2018). Association between Loss of Skeletal Muscle Mass and Mortality and Tumor Recurrence in Hepatocellular Carcinoma: A Systematic Review and Meta-Analysis. Liver Cancer.

[5] Bokshan S.L., DePasse J.M., Daniels A.H. (2016). Sarcopenia in Orthopedic Surgery. Orthopedics.

[6] Boutin R.D., Bamrungchart S., Bateni C.P., Beavers D.P., Beavers K.M., Meehan J.P., Lenchik L. (2017). CT of Patients with Hip Fracture: Muscle Size and Attenuation Help Predict Mortality. Am. J. Roentgenol..

[7] Friedman J., Lussiez A., Sullivan J., Wang S., Englesbe M. (2015). Implications of Sarcopenia in Major Surgery. Nutr. Clin. Pract..

[8] Dirks R.C., Edwards B.L., Tong E., Schaheen B., Turrentine F.E., Shada A., Smith P.W. (2017). Sarcopenia in emergency abdominal surgery. J. Surg. Res..

[9] Schiaffino S., Albano D., Cozzi A., Messina C., Arioli R., Bnà C., Bruno A., Carbonaro L.A., Carriero A., Carriero S. (2021). CT-derived Chest Muscle Metrics for Outcome Prediction in Patients with COVID-19. Radiology.

[10] Albano D., Messina C., Vitale J., Sconfienza L.M. (2020). Imaging of sarcopenia: Old evidence and new insights. Eur. Radiol..

[11] Messina C., Albano D., Gitto S., Tofanelli L., Bazzocchi A., Ulivieri F.M., Guglielmi G., Sconfienza L.M. (2020). Body composition with dual energy X-ray absorptiometry: From basics to new tools. Quant. Imaging Med. Surg..

[12] Chianca V., Albano D., Messina C., Gitto S., Ruffo G., Guarino S., Del Grande F., Sconfienza L.M. (2021). Sarcopenia: Imaging assessment and clinical application. Abdom. Radiol..

[13] Cruz-Jentoft A.J., Bahat G., Bauer J., Boirie Y., Bruyère O., Cederholm T., Cooper C., Landi F., Rolland Y., Sayer A.A. (2019). Sarcopenia: Revised European consensus on definition and diagnosis. Age Ageing.

[14] Boutin R.D., Yao L., Canter R.J., Lenchik L. (2015). Sarcopenia: Current Concepts and Imaging Implications. Am. J. Roentgenol..

[15] Shen W., Punyanitya M., Wang Z., Gallagher D., St.-Onge M.-P., Albu J., Heymsfield S.B., Heshka S. (2004). Total body skeletal muscle and adipose tissue volumes: Estimation from a single abdominal cross-sectional image. J. Appl. Physiol..

[16] Tuttle C.S., Thang L.A., Maier A.B. (2020). Markers of inflammation and their association with muscle strength and mass: A systematic review and meta-analysis. Ageing Res. Rev..

[17] Zakynthinos E., Pappa N. (2009). Inflammatory biomarkers in coronary artery disease. J. Cardiol..

[18] Miceli G., Basso M.G., Rizzo G., Pintus C., Tuttolomondo A. (2022). The Role of the Coagulation System in Peripheral Arterial Disease: Interactions with the Arterial Wall and Its Vascular Microenvironment and Implications for Rational Therapies. Int. J. Mol. Sci..

[19] Miceli G., Basso M.G., Pintus C., Pennacchio A.R., Cocciola E., Cuffaro M., Profita M., Rizzo G., Tuttolomondo A. (2024). Molecular Pathways of Vulnerable Carotid Plaques at Risk of Ischemic Stroke: A Narrative Review. Int. J. Mol. Sci..

[20] Cury R.C., Leipsic J., Abbara S., Achenbach S., Berman D., Bittencourt M., Budoff M., Chinnaiyan K., Choi A.D., Ghoshhajra B. (2022). CAD-RADS™ 2.0-2022 Coronary Artery Disease-Reporting and Data System. J. Cardiovasc. Comput. Tomogr..

[21] Mergen V., Ried E., Allmendinger T., Sartoretti T., Higashigaito K., Manka R., Euler A., Alkadhi H., Eberhard M. (2022). Epicardial Adipose Tissue Attenuation and Fat Attenuation Index: Phantom Study and In Vivo Measurements with Photon-Counting Detector CT. Am. J. Roentgenol..

[22] La Grutta L., Toia P., Farruggia A., Albano D., Grassedonio E., Palmeri A., Maffei E., Galia M., Vitabile S., Cademartiri F. (2016). Quantification of epicardial adipose tissue in coronary calcium score and CT coronary angiography image data sets: Comparison of attenuation values, thickness and volumes. Br. J. Radiol..

[23] Antonopoulos A.S., Sanna F., Sabharwal N., Thomas S., Oikonomou E.K., Herdman L., Margaritis M., Shirodaria C., Kampoli A.-M., Akoumianakis I. (2017). Detecting human coronary inflammation by imaging perivascular fat. Sci. Transl. Med..

[24] Oikonomou E., Marwan M., Desai M.Y., Mancio J., Alashi A., Centeno E.H., Thomas S., Herdman L., Kotanidis C., Thomas K.E. (2018). Non-invasive detection of coronary inflammation using computed tomography and prediction of residual cardiovascular risk (the CRISP CT study): A post-hoc analysis of prospective outcome data. Lancet.

[25] Erkan M., Zengin I., Bekircavuşoğlu S., Topal D., Bulut T., Erkan H. (2023). Effect of Sarcopenia on Coronary Atherosclerotic Burden, Lesion Complexity, and Major Cardiovascular Events in Elderly Patients With Acute Coronary Syndrome: A 1-year Follow-up Study. Angiology.

[26] Dvoretskiy S., Lieblein-Boff J.C., Jonnalagadda S., Atherton P.J., Phillips B.E., Pereira S.L. (2020). Exploring the Association between Vascular Dysfunction and Skeletal Muscle Mass, Strength and Function in Healthy Adults: A Systematic Review. Nutrients.

[27] Amarasekera A.T., Chang D., Schwarz P., Tan T.C. (2021). Does vascular endothelial dysfunction play a role in physical frailty and sarcopenia? A systematic review. Age and Ageing.

[28] Shin J.Y., Lim J.S. (2021). Muscle mass and grip strength in relation to carotid intima-media thickness and plaque score in patients with type 2 diabetes. Nutr. Metab. Cardiovasc. Dis..

[29] Lee H.S., Park K.W., Kang J., Ki Y.-J., Chang M., Han J.-K., Yang H.-M., Kang H.-J., Koo B.-K., Kim H.-S. (2020). Sarcopenia Index as a Predictor of Clinical Outcomes in Older Patients with Coronary Artery Disease. J. Clin. Med..

[30] Jun J.E., Choi M.S., Park S.W., Kim G., Jin S.-M., Kim K., Hwang Y.-C., Ahn K.J., Chung H.Y., Jeong I.-K. (2021). Low Skeletal Muscle Mass Is Associated With the Presence, Incidence, and Progression of Coronary Artery Calcification. Can. J. Cardiol..

[31] Zannoni S., Albano D., Jannone M.L., Messina C., Sconfienza L.M. (2020). Correlation between muscle mass and quality around the hip and of psoas muscles at L3 level using unenhanced CT scans. Skelet. Radiol..

[32] Albano D., Gitto S., Vitale J., Bernareggi S., Aliprandi A., Sconfienza L.M., Messina C. (2022). Comparison between magnetic resonance imaging and electrical impedance myography for evaluating lumbar skeletal muscle composition. BMC Musculoskelet. Disord..

[33] Cury R.C., Leipsic J., Abbara S., Achenbach S., Berman D., Bittencourt M., Budoff M., Chinnaiyan K., Choi A.D., Ghoshhajra B. (2022). CAD-RADS™ 2.0-2022 Coronary Artery Disease—Reporting and Data System An Expert Consensus Document of the Society of Cardiovascular Computed Tomography (SCCT), the American College of Cardiology (ACC), the American College of Radiology (ACR) and the North America Society of Cardiovascular Imaging (NASCI). Radiol. Cardiothorac. Imaging.

[34] Di Leo G., Sardanelli F. (2020). Statistical significance: P value, 0.05 threshold, and applications to radiomics—Reasons for a conservative approach. Eur. Radiol. Exp..

[35] Lee G.K.-Y., Au P.C.-M., Li G.H.-Y., Chan M., Li H.-L., Cheung B.M.-Y., Wong I.C.-K., Lee V.H.-F., Mok J., Yip B.H.-K. (2021). Sarcopenia and mortality in different clinical conditions: A meta-analysis. Osteoporos. Sarcopenia.

[36] (2022). The DISCHARGE Trial Group CT or Invasive Coronary Angiography in Stable Chest Pain. N. Engl. J. Med..

[37] Zhang R., Ju Z., Li Y., Gao Y., Gu H., Wang X. (2022). Pericoronary fat attenuation index is associated with plaque parameters and stenosis severity in patients with acute coronary syndrome: A cross-sectional study. J. Thorac. Dis..

[38] Monti C.B., Codari M., De Cecco C.N., Secchi F., Sardanelli F., Stillman A.E. (2019). Novel imaging biomarkers: Epicardial adipose tissue evaluation. Br. J. Radiol..

[39] Iacobellis G. (2021). Aging Effects on Epicardial Adipose Tissue. Front. Aging.

[40] Doukbi E., Soghomonian A., Sengenès C., Ahmed S., Ancel P., Dutour A., Gaborit B. (2022). Browning Epicardial Adipose Tissue: Friend or Foe?. Cells.

[41] Go S.-I., Park M.J., Song H.-N., Kim H.-G., Kang M.H., Kang J.H., Kim H.R., Lee G.-W. (2017). A comparison of pectoralis versus lumbar skeletal muscle indices for defining sarcopenia in diffuse large B-cell lymphoma-two are better than one. Oncotarget.

[42] Wilson P.W.F., D’Agostino R.B., Levy D., Belanger A.M., Silbershatz H., Kannel W.B. (1998). Prediction of Coronary Heart Disease Using Risk Factor Categories. Circulation.

[43] Rahimi K., Bidel Z., Nazarzadeh M., Copland E., Canoy D., Wamil M., Majert J., McManus R., Adler A., Agodoa L. (2021). Age-stratified and blood-pressure-stratified effects of blood-pressure-lowering pharmacotherapy for the prevention of cardiovascular disease and death: An individual participant-level data meta-analysis. Lancet.

[44] Hathaway Q., Ibad H.A., Bluemke D.A., Pishgar F., Kasaiean A., Klein J.G., Cogswell R., Allison M., Budoff M.J., Barr R.G. (2023). Predictive Value of Deep Learning–derived CT Pectoralis Muscle and Adipose Measurements for Incident Heart Failure: Multi-Ethnic Study of Atherosclerosis. Radiol. Cardiothorac. Imaging.

[45] Teigen L.M., John R., Kuchnia A.J., Nagel E.M., Earthman C.P., Kealhofer J., Martin C., Cogswell R. (2017). Preoperative Pectoralis Muscle Quantity and Attenuation by Computed Tomography Are Novel and Powerful Predictors of Mortality After Left Ventricular Assist Device Implantation. Circ. Heart Fail..

[46] Damluji A.A., Alfaraidhy M., AlHajri N., Rohant N.N., Kumar M., Al Malouf C., Bahrainy S., Kwak M.J., Batchelor W.B., Forman D.E. (2023). Sarcopenia and Cardiovascular Diseases. Circulation.

[47] Gallone G., Depaoli A., D’Ascenzo F., Tore D., Allois L., Bruno F., Casale M., Atzeni F., De Lio G., Bocchino P.P. (2022). Impact of computed-tomography defined sarcopenia on outcomes of older adults undergoing transcatheter aortic valve implantation. J. Cardiovasc. Comput. Tomogr..

[48] Phan E.N., Thorpe S.W., Wong F.S., Saiz A.M., Taylor S.L., Canter R.J., Lenchik L., Randall R.L., Boutin R.D. (2020). Opportunistic muscle measurements on staging chest CT for extremity and truncal soft tissue sarcoma are associated with survival. J. Surg. Oncol..

[49] Yao L., Petrosyan A., Chaudhari A.J., Lenchik L., Boutin R.D. (2024). Clinical, functional, and opportunistic CT metrics of sarcopenia at the point of imaging care: Analysis of all-cause mortality. Skelet. Radiol..

